# Association between physical performance and bone mass in community-dwelling postmenopausal Japanese women: The Unzen study

**DOI:** 10.1371/journal.pone.0296457

**Published:** 2024-01-02

**Authors:** Masahiro Suiko, Satoshi Mizukami, Kazuhiko Arima, Hiroki Nakashima, Takayuki Nishimura, Yoshihito Tomita, Yasuyo Abe, Natsumi Tanaka, Yuzo Honda, Michiko Kojima, Tetsuji Okawachi, Maiko Hasegawa, Youko Sou, Ritsu Tsujimoto, Mitsuo Kanagae, Makoto Osaki, Kiyoshi Aoyagi

**Affiliations:** 1 Department of Public Health, Nagasaki University Graduate School of Biomedical Sciences, Nagasaki, Japan; 2 Department of Orthopedic Surgery, Nagasaki University Graduate School of Biomedical Sciences, Nagasaki, Japan; 3 Leading Medical Research Core Unit, Nagasaki University Graduate School of Biomedical Sciences, Nagasaki, Japan; 4 Department of Human Science, Faculty of Design, Kyushu University, Fukuoka, Japan; 5 Department of Physical Therapy, School of Rehabilitation, Tokyo Professional University of Health Science, Tokyo, Japan; 6 Department of Health and Nutrition Science, Nishikyusyu University, Kanzaki, Japan; 7 Department of Nursing, Nishikyusyu University, Ogi, Japan; 8 Medical Policy Division, Nagasaki Prefectural Government, Nagasaki, Japan; 9 Ken-Nan Health Care Office, Nagasaki, Japan; 10 Department of Rehabilitation, Nishi-Isahaya Hospital, Isahaya, Japan; King Abdulaziz University Faculty of Medicine, SAUDI ARABIA

## Abstract

**Background:**

Low bone mass is an independent risk factor for osteoporotic fractures. We examined the association between physical performance and bone mass using quantitative ultrasound in community-dwelling postmenopausal Japanese women.

**Methods:**

We conducted a cross-sectional study on 524 community-dwelling postmenopausal Japanese women who were not being administered osteoporosis medications. Physical performance was assessed on the basis of grip strength, chair stand time, and functional reach. The stiffness index was measured as a quantitative ultrasound parameter for heel bone mass.

**Results:**

Physical performance, assessed by grip strength, chair stand time, and functional reach, and the stiffness index significantly decreased with age (both p<0.001). The multiple linear regression analysis showed that grip strength (p = 0.001), chair stand time (p = 0.004), and functional reach (p = 0.048) were significantly associated with the stiffness index after adjusting for age, body mass index, smoking, drinking, and exercise.

**Conclusions:**

Physical performance was significantly associated with heel bone mass in community-dwelling postmenopausal Japanese women.

## Introduction

Osteoporosis is a systemic skeletal disease characterized by low bone mass and microarchitectural deterioration of bone tissue, that leads to enhanced bone fragility and a consequent increase in fracture risk [1]. Osteoporosis is associated with a fracture risk [2], functional disability, reduced quality of life, higher health-care costs [3, 4], and increased mortality risk [5]. Owing to the rapid aging of the population, the prevalence of osteoporosis and osteoporotic fractures is increasing, especially among Asians, who are reported to be at higher risk for osteoporosis-related fractures with an estimated >50% of all osteoporotic fractures occurring in Asian women by 2050 [6]. Therefore, preventing osteoporosis and low bone mass is an important issue.

In the older population, the risk of fragility fractures resulting from falls is high. Physical performance measures can predict the risk and subsequent consequences of falls [7]. Dai et al. [8] reported that lower-extremity muscle performance is associated with estimated fracture risk in community-dwelling postmenopausal women. Therefore, the measurement of physical performance is important for assessing fracture risk.

Quantitative ultrasound (QUS) provides useful information for bone mass assessment [9]. The International Society for Clinical Densitometry guidelines indicate that the heel is the only validated skeletal site for the clinical use of QUS in osteoporosis management [10]. Although the diagnosis of osteoporosis is commonly based on measurements taken using dual-energy X-ray absorptiometry (DXA) [11, 12], QUS is widely used for the screening of osteoporosis because it is free of ionizing radiation, portable, easy to use, and inexpensive [13]. Large prospective studies have confirmed that both speed of sound (SOS) and broadband ultrasound attenuation (BUA) measurements in heel QUS can identify individuals at risk of osteoporotic fractures as reliably as bone mineral density (BMD) [14, 15]. Trimpou et al. [16] also showed that the stiffness index measured by QUS was positively correlated with BMD measured by DXA in each region (total, femoral neck, and lumbar spine). Therefore, QUS is a useful tool for preliminary screening for osteoporosis [17–20].

Lower physical performance and BMD or bone mass are considered risk factors for fractures. Some studies have reported that physical performance has a weak or no association with BMD at various skeletal sites [21, 22], whereas other studies have reported significant associations between physical performance and BMD or bone mass [23–25]. Therefore, the relationship between physical performance and BMD or bone mass is unclear.

Exercise may influence physical performance and bone mass; however, its role remains unclear [26]. Further, exercise may be a safe and effective way to avert bone loss in postmenopausal women; however, physical performance and bone mass may change independently of exercise due to age and lifestyle effects [27]. Several studies have suggested that smoking and alcohol consumption are associated with bone loss. [28, 29].

Thus, this study aimed to examine the association between physical performance and bone mass as measured by QUS after adjusting for covariates in community-dwelling postmenopausal Japanese women.

## Materials and methods

### Subjects

**Fig 1:**
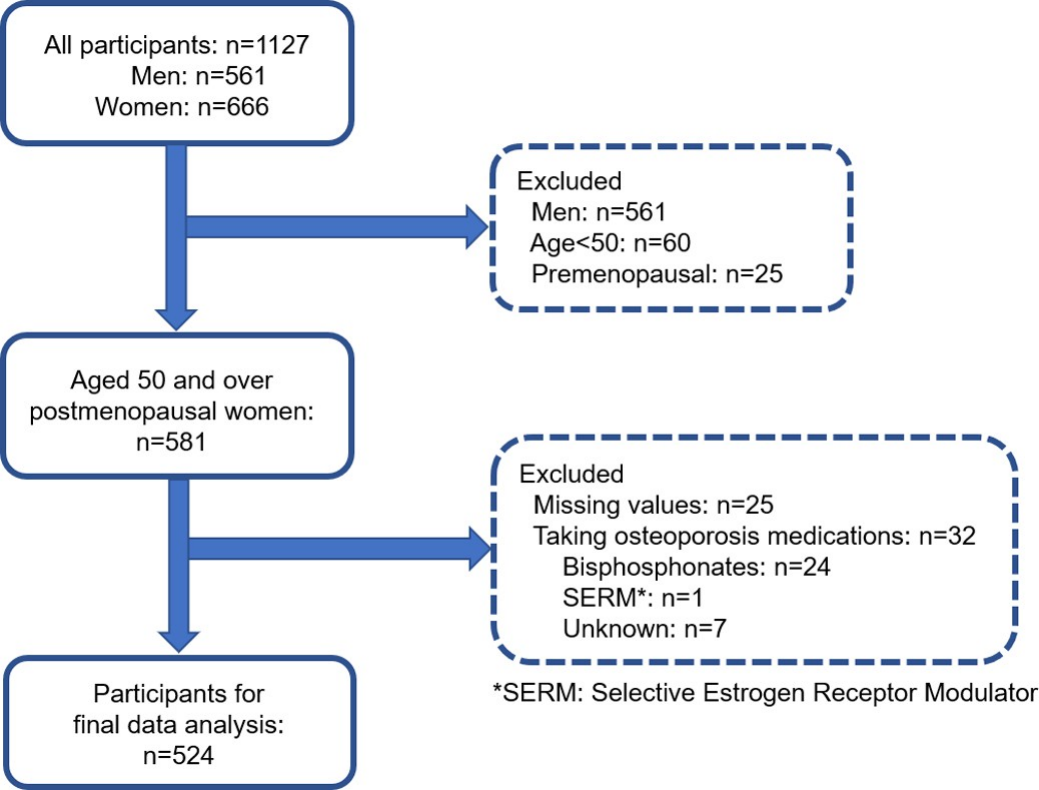
Flowchart of participant selection.

A cross-sectional study, the Unzen study, was conducted including 1127 community-dwelling Japanese adults (561 men and 666 women) who reside in Unzen city, Japan [30]. Study participants were recruited among individuals who underwent annual health examinations designed for lifestyle health check-ups and health guidance between May 11, 2011 and November 1, 2013 and were limited to postmenopausal women aged 50 years and older. Postmenopausal status was defined as menopause at least 1 year after the last menstruation. Participants with missing values (n = 25) for any variables or those taking osteoporosis medications (n = 32) were excluded from the analysis, with the remaining participants (n = 524) included in the final data analysis (Fig 1).

All participants provided written informed consent before examinations. This study was approved by the Ethics Committee of Nagasaki University Graduate School of Biomedical Sciences (No. 11072739) and adhered to the Strengthening the Reporting of Observational Studies in Epidemiology (STROBE) Statement [31].

### Measurements

Height (cm) and weight (kg) were measured while wearing light clothing and no shoes, and the body mass index (BMI) was calculated as weight/height squared (kg/m^2^). Information on menopause age, current smoking (yes/no), alcohol consumption (heavy drinking, ≥20 g/day; moderate drinking, 0<, <20 g/day; and non-drinking), and exercise (at least 30 min twice per week) was collected by interview.

Grip strength was measured using a hydraulic hand dynamometer (Jamar Hydraulic Hand Dynamometer 5030 J1; Jafayette Instrument Company, Inc., Jafayette, IN, USA). The participant’s dominant hand was considered for the analysis. The chair stand time was measured as the time taken to stand up from a standard chair five times; if possible, the subjects were asked not to use their arms for assistance. Functional reach was calculated as the difference between two measurements. The participants first stood, facing forward, hand in a fist, with their arm extended next to a yardstick mounted on a wall, and subsequently reached forward as far as possible without stepping or losing their balance.

All physical performance measures were obtained twice, and their average values were analyzed [32–34]. The heel QUS parameters, including speed of sound (SOS), broadband ultrasound (BUA), and stiffness index (0.67 x BUA + 0.28 x SOS ‐ 420), were measured only once using a Lunar Achilles device (Achilles InSight GE Lunar Corp., Madison, WI). In all participants, QUS parameters were measured on the right heel. In this study, the stiffness index was adopted as the index of bone mass. Measurements of all physical performance measures and QUS parameters were performed by several trained health-care professionals (e.g., physicians, nurses, physical therapists, and occupational therapists) on the same day. T-score of stiffness index were used to classify participants into three groups: "-1.0 ≤ T-score ", "-2.5 < T-score < -1.0” and “T-score ≤ -2.5", according to the definition for osteoporosis using T-score recommended by the WHO.

### Statistical analysis

Data were analyzed using SPSS statistical software version 27 (SPSS, Inc., Chicago, IL). The averages physical performance and stiffness index among the 10-year age groups were analyzed using analysis of variance (ANOVA) to identify differences between each group, whereas the trends were analyzed using general linear modeling methods to identify age-related differences. The null hypothesis for ANOVA is that all population means are equal [35]. Age-adjusted means of physical performance measures and stiffness indices between women with and without exercise were analyzed by analysis of covariance (ANCOVA) using general linear modeling methods. The null hypothesis for ANCOVA is that all population means are equal when controlling for 1+ covariates [35]. The multiple linear regression analysis was used to explore the association between physical performance and the stiffness index after adjusting for age, BMI, current smoking, alcohol consumption, and exercise. Statistical *p* value of less than 0.05 was considered significant.

## Results

The participants’ characteristics are shown in Table 1. The mean age was 67.4 ± 7.2 years. The prevalence rates of smoking and heavy alcohol consumption were 1.1% and 1.3%, respectively. One-third of the participants indulged in exercise.

**Table 1:** Characteristics of subjects (n = 524).

Characteristics	Mean (SD)
Age (years)	67.4±7.2
Height (cm)	151.2±5.6
Weight (kg)	51.0±8.1
Body mass index (kg/m^2^)	22.3±3.2
Time since menopause (years)	17.4±8.9
Grip strength (kg)	23.8±5.2
Chair stand time (sec)	7.5±2.0
Functional reach (cm)	32.4±6.8
Stiffness index	68.7±13.0
	n (%)
T-score of stiffness index	
-1.0 ≤	70 (13.3)
-2.5 <, < -1.0	209 (39.9)
≤ -2.5	245 (46.8)
Exercise	
with	168 (32.1)
without	356 (67.9)
Smoking	
Non-smoking	518 (98.9)
Current smoking	6 (1.1)
Alcohol consumption	
Non-drinking	455 (86.8)
Moderate drinking (0<, <20g/day)	62 (11.8)
Heavy drinking (≥ 20 g/day)	7 (1.3)

Data are shown as means±standard deviation or n (%).

SD: standard deviation

Table 2 shows age-specific mean (SD) of the physical performance measures and the stiffness index. Physical functioning decreased (grip strength decreased, chair stand time increased, and functional reach decreased) with age (p<0.001). The stiffness index significantly decreased with age (p<0.001).

**Table 2:** Age-specific mean (SD) of physical performance measures and the stiffness index.

Age group	50–59	60–69	70–79	80-	ANOVA	Trend
(years)	(n = 85)	(n = 228)	(n = 184)	(n = 27)		
Grip strength (kg)	26.3 (4.4)	24.9 (4.7)	21.8 (4.9)	18.8 (6.5)	<0.001	<0.001
Chair stand time (sec)	6.6 (1.4)	7.1 (1.5)	8.1 (2.3)	9.6 (2.9)	<0.001	<0.001
Functional reach (cm)	35.2 (7.0)	33.2 (5.7)	31.0 (7.1)	26.5 (6.2)	<0.001	<0.001
Stiffness index	73.8(11.4)	72.0 (12.7)	63.8 (12.0)	58.9 (9.7)	<0.001	<0.001

One-way analysis of variance (ANOVA) and linear regression analysis.

Table 3 shows the age-adjusted mean (SE) of the physical performance measures and the stiffness index by status of exercise, smoking, and alcohol consumption. Functional reach and stiffness index were significantly higher in those who exercised than those in those who did not. However, there were no significant differences in physical performance measures and the stiffness index by smoking and alcohol consumption.

**Table 3:** Comparison of age-adjusted mean (SE) of physical performance measures and stiffness index by status of exercise, smoking, and alcohol consumption.

	Exercise			Smoking			Alcohol consumption			
	With	Without	p value	Non	Current	p value	Non	Moderate	Heavy	p value
	n = 168	n = 356		n = 518	n = 6		n = 455	n = 62	n = 7	
Grip strength (kg)	24.0 (0.4)	23.6 (0.3)	0.47	23.7 (0.2)	24.8 (2.0)	0.58	23.8 (0.2)	23.4 (0.6)	26.1 (1.8)	0.36
Chair stand time (sec)	7.3 (0.1)	7.6 (0.1)	0.07	7.5 (0.1)	8.3 (0.8)	0.32	7.5 (0.1)	7.6 (0.2)	6.1 (0.7)	0.15
Functional reach (cm)	33.4 (0.5)	31.9 (0.3)	0.02	32.4 (0.3)	30.5 (2.6)	0.47	32.4 (0.3)	32.3 (0.8)	35.1 (2.4)	0.53
Stiffness index	71.3 (0.9)	67.5 (0.6)	0.001	68.8 (0.5)	65.1 (5.0)	0.47	68.9 (0.6)	68.5 (1.5)	62.0 (4.6)	0.32

Data are shown as age-adjusted means (standard errors) using analysis of covariance (ANCOVA).

Table 4 shows the association between physical performance measures and the stiffness index in multiple linear regression analysis adjusted for covariates (age, BMI, current smoking, alcohol consumption, and exercise). Grip strength (p = 0.001), chair stand time (p = 0.004), and functional reach (p = 0.048) were significantly associated with the stiffness index after adjusting for age, BMI, smoking, alcohol consumption, and exercise.

**Table 4:** Association between physical performance measures and the stiffness index in multiple linear regression models.

	Crude		Age-adjusted		Age, BMI, smoking, alcohol consumption and exercise-adjusted	
	B (SE)	p value	B (SE)	p value	B (SE)	p value
Grip strength (kg)	0.7 (0.1)	<0.001	0.4 (0.1)	<0.001	0.4 (0.1)	0.001
Chair stand time (sec)	-1.6 (0.3)	<0.001	-0.8 (0.3)	0.004	-0.8 (0.3)	0.003
Functional reach (cm)	0.3 (0.1)	<0.001	0.1 (0.1)	0.11	0.2 (0.1)	0.048

Multiple linear regression analysis

B: partial regression coefficient

SE: standard error

## Discussion

We examined the association between physical performance and bone mass by QUS in community-dwelling postmenopausal Japanese women. Grip strength, chair stand time and functional reach were significantly associated with the stiffness index after adjusting for age, BMI, smoking, alcohol consumption, and exercise. Previous studies have shown significant associations between physical performance measures and bone mass by QUS [23, 24], which are consistent with our results.

Heel bone mass (stiffness index) decreased significantly with age in postmenopausal women. Consistent with our results, previous studies have reported that aging is significantly associated with a lower stiffness index in postmenopausal women [24, 36, 37].

Similar to the results of previous reports, we found that physical performance decreased significantly with age in postmenopausal women [25, 38–43]. Grip strength primarily measures upper body appendicular muscle strength [44–48], and chair stand time is significantly associated with knee extensor muscle strength [49]. Muscle strength and physical performance significantly decline with age, and muscle strength positively correlates with muscle mass [50]. Muscle mass declines by 1.5% per year after the age of 50 years and by 3% per year after the age of 80 years [51]. Therefore, exercise interventions that promote the maintenance or increase of muscle strength and mass may be necessary to prevent age-related declines in physical performance.

We found a significantly higher heel bone mass in women who exercised than in those who did not. Zhang et al. [52] reported that higher physical activity is significantly associated with a higher stiffness index in postmenopausal women. Yung et al. [53] reported that the performance of weight-bearing exercises was associated with increased bone mass compared to that associated with the performance of non-weight-bearing exercises. Osteoblasts produce new bone tissue when the daily mechanical load exceeds normal physiological levels, while osteoclasts remove existing bone tissue when the daily mechanical load falls below normal levels [54]. Previous reports have suggested that microgravity contributes to bone loss [55]. Although we did not evaluate the content of the exercise, mechanical stress that is produced by exercise may contribute to good bone health.

Grip strength was significantly and positively associated with QUS parameters (SOS and stiffness index) [23, 24], which is consistent with our results. Kritz et al. [56] reported that grip strength was significantly associated with BMD at the radius, lumbar spine, and hip joint, and Vogel et al. [57] reported that calcaneal BMD correlated with radius, spine, and hip BMD. Grip strength has also been reported to be positively correlated with the muscle function of the lower extremities and the knee-extension strength [58, 59]. Therefore, although grip strength does not directly reflect the force on the heel, it may be indirectly related to heel bone mass.

In this study, chair stand time was significantly and negatively associated with the stiffness index. Taaffe et al. [60] reported that chair stand time is significantly associated with hip BMD. Dai et al. [8] reported that the short physical performance battery score, which reflects lower extremity function including chair rise time, was significantly associated with hip BMD. The chair stand, which reflects leg muscle strength, directly affects the calcaneus owing to the weight-bearing exercise load and may be related to bone mass.

In this study, functional reach was significantly positively associated with the stiffness index. Functional reach has been used to assess balance impairment [39]. Aoyagi et al. [25] reported a significant association between functional reach and BMD using single-energy X-ray absorptiometry. Taaffe et al. [60] assessed the balance function in terms of the total time spent in a semi-tandem stand, tandem stand, and one-leg stand, and reported a significant association with hip BMD in older black women. Similarly, Lindsey et al. [61] reported a significant association between one-leg stand time and hip and whole-body BMD in postmenopausal women; they also noted that the association between one-leg stand time and BMD was influenced by muscle power, because one-leg stand time correlated with measures of physical performance in the upper and lower extremities. Although a positive association was found between functional reach and the stiffness index in this study (p = 0.048), to the best of our knowledge, no study has reported an association between functional reach and heel bone mass using QUS. Further research is required to explore the association between balance tests and QUS parameters.

This study had several limitations. First, because we used a cross-sectional design, we could not establish causal relationships between the physical performance measures and bone mass by QUS. A longitudinal study is required to determine causality. Second, there is a possibility of selection bias because our participants underwent periodic health examinations. Third, we could not assess other factors that could potentially influence bone health such as nutrition, occupation, former smoking history, and calcium and vitamin D supplementation history. Fourth, because we assessed bone mass by QUS and did not measure BMD by DXA or quantitative computed tomography (QCT), the findings herein cannot be compared directly with those of a previous study that measured BMD using DXA or QCT. Fifth, we could not assess the participants’ history of osteoporotic fractures that may affect bone metabolism.

## Conclusion

In the present study, physical performance measures (grip strength, chair stand time, and functional reach) were significantly associated with heel bone mass in community-dwelling postmenopausal Japanese women.

## Supporting information

S1 Dataset (CSV)
